# SGLFormer: Spiking Global-Local-Fusion Transformer with high performance

**DOI:** 10.3389/fnins.2024.1371290

**Published:** 2024-03-12

**Authors:** Han Zhang, Chenlin Zhou, Liutao Yu, Liwei Huang, Zhengyu Ma, Xiaopeng Fan, Huihui Zhou, Yonghong Tian

**Affiliations:** ^1^AI Department, Peng Cheng Laboratory, Shenzhen, China; ^2^Faculty of Computing, Harbin Institute of Technology, Harbin, China; ^3^National Key Laboratory for Multimedia Information Processing, School of Computer Science, Peking University, Beijing, China

**Keywords:** Spiking Neural Network, spiking transformer, Global-Local-Fusion, Maxpooling, spatio-temporal, high performance

## Abstract

**Introduction:**

Spiking Neural Networks (SNNs), inspired by brain science, offer low energy consumption and high biological plausibility with their event-driven nature. However, the current SNNs are still suffering from insufficient performance.

**Methods:**

Recognizing the brain's adeptness at information processing for various scenarios with complex neuronal connections within and across regions, as well as specialized neuronal architectures for specific functions, we propose a Spiking Global-Local-Fusion Transformer (SGLFormer), that significantly improves the performance of SNNs. This novel architecture enables efficient information processing on both global and local scales, by integrating transformer and convolution structures in SNNs. In addition, we uncover the problem of inaccurate gradient backpropagation caused by Maxpooling in SNNs and address it by developing a new Maxpooling module. Furthermore, we adopt spatio-temporal block (STB) in the classification head instead of global average pooling, facilitating the aggregation of spatial and temporal features.

**Results:**

SGLFormer demonstrates its superior performance on static datasets such as CIFAR10/CIFAR100, and ImageNet, as well as dynamic vision sensor (DVS) datasets including CIFAR10-DVS and DVS128-Gesture. Notably, on ImageNet, SGLFormer achieves a top-1 accuracy of 83.73% with 64 M parameters, outperforming the current SOTA directly trained SNNs by a margin of 6.66%.

**Discussion:**

With its high performance, SGLFormer can support more computer vision tasks in the future. The codes for this study can be found in https://github.com/ZhangHanN1/SGLFormer.

## 1 Introduction

Inspired by brain science, Spiking Neural Networks (SNNs) use binary spikes to transmit information, which are event-driven, and offer low energy consumption and high biological plausibility. SNNs are regarded as the next generation of neural networks (Maass, 1997). However, current SNNs are still suffering from insufficient performance. Complex patterns of neuronal connections within and across brain regions, along with specialized neuronal architectures for particular functions, enable the brain to adeptly handle information processing across diverse scenarios (Luo, 2021). Information processing in visual pathways can be modeled by convolutional structure (Fukushima, 1980). In visual pathways, a neuron receives spikes from presynaptic neurons within its receptive field and processes the incoming information in the soma. However, the difference is that the process of convolution operation in Convolutional Neural Networks (CNNs) is analogous. The receptive field in CNNs is determined by the size of the convolution kernel. Recent researches indicate that certain neuronal connections share functional similarities with the transformer architecture (Vaswani et al., 2017). Specifically, a network composed of astrocytes, neurons, and tripartite synapses between them, was proven to naturally implement the core operations of transformer structure (Kozachkov et al., 2023). Moreover, a recent study revealed that the transformer functions are similar to the hippocampus, when equipped with recursive positional encoding, the transformer structure can accurately replicate the spatial representation of hippocampal formation (Whittington et al., 2021). Integrating convolutional and transformer structures in SNNs can help process both local and global information simultaneously, potentially improving the performance of SNNs. In the realm of artificial neural networks (ANNs), a similar idea was adopted in various models (Chen et al., 2022; Guo et al., 2022; Peng et al., 2023).

Convolution-based SNNs are suitable for vision tasks, with their inherent translational invariance and inductive bias. However, the training of SNNs is challenging due to the non-differentiable nature of their activation functions. Surrogate Gradient (SG) method (Neftci et al., 2019), replacing the original non-differentiable step function in neurons with a differentiable function during backpropagation, simplified the training of convolution-based SNNs. Gradient vanishing and explosion significantly impede the scaling and performance enhancement of SNNs. The application of residual connections and some normalization techniques (He et al., 2015; Fang et al., 2021a; Zheng et al., 2021; Hu et al., 2023a) demonstrated substantial effectiveness in enhancing network depth and performance. Specifically, threshold-dependent batch normalization (tdBN; Zheng et al., 2021) was proposed to alleviate the problems of gradient vanishing and explosion, and successfully applied to train convolution-based SNNs up to 50 layers. Furthermore, Spike-Element-wise (SEW) ResNet (Fang et al., 2021a) further mitigated the gradient vanishing and explosion problem, obtaining a directly trained SNN beyond 100 layers for the first time, and achieved notable top-1 accuracy on the ImageNet dataset. Despite these breakthroughs, Convolution-based SNNs still suffer to meet the evolving demands of complex computational tasks.

Vision Transformer (ViT; Dosovitskiy et al., 2020) showed superior performance in a wide range of vision tasks. This success led to increased interest in integrating transformer architectures with SNNs. Spikeformer (Li et al., 2022) utilized spatial-temporal self-attention to extract global features in both spatial and temporal domains. However, Spikeformer struggled with a high computational load due to numerous floating-point multiplications and exponential operations in softmax. Spikformer (Zhou Z. et al., 2023) proposed spiking self-attention (SSA), innovatively eliminating the softmax function, and reducing computational complexity while enhancing performance. Moreover, based on the Spikformer, other spiking transformers were developed to improve the performance, such as Spikingformer (Zhou C. et al., 2023) and Spike-driven Transformer (Yao et al., 2023a). However, there remains a performance gap when comparing spiking transformers to their ANN counterparts, suggesting an ongoing opportunity for further development.

In this study, to address the challenge of limited performance, we propose a directly trained SNN that integrates convolutional structure and transformer structure, named Spiking Global-Local-Fusion Transformer (SGLFormer). In addition, we uncover the issue of inaccurate gradient backpropagation induced by inappropriate Maxpooling operations in SNNs, which hampers the performance. This is addressed by the development of an SNN-optimized Maxpooling module. Moreover, the spatio-temporal block (STB) is employed in the classification head to aggregate spatial and temporal features effectively. Experimental results show that various components of SGLFormer collaboratively contribute to improving its performance. SGLFormer achieves high performance on both static and dynamic vision sensor (DVS) datasets, especially on ImageNet, with a top-1 accuracy of 83.73%, significantly surpassing existing SOTA methods.

## 2 Method

### 2.1 The overall framework of SGLFormer

**Figure 1:**
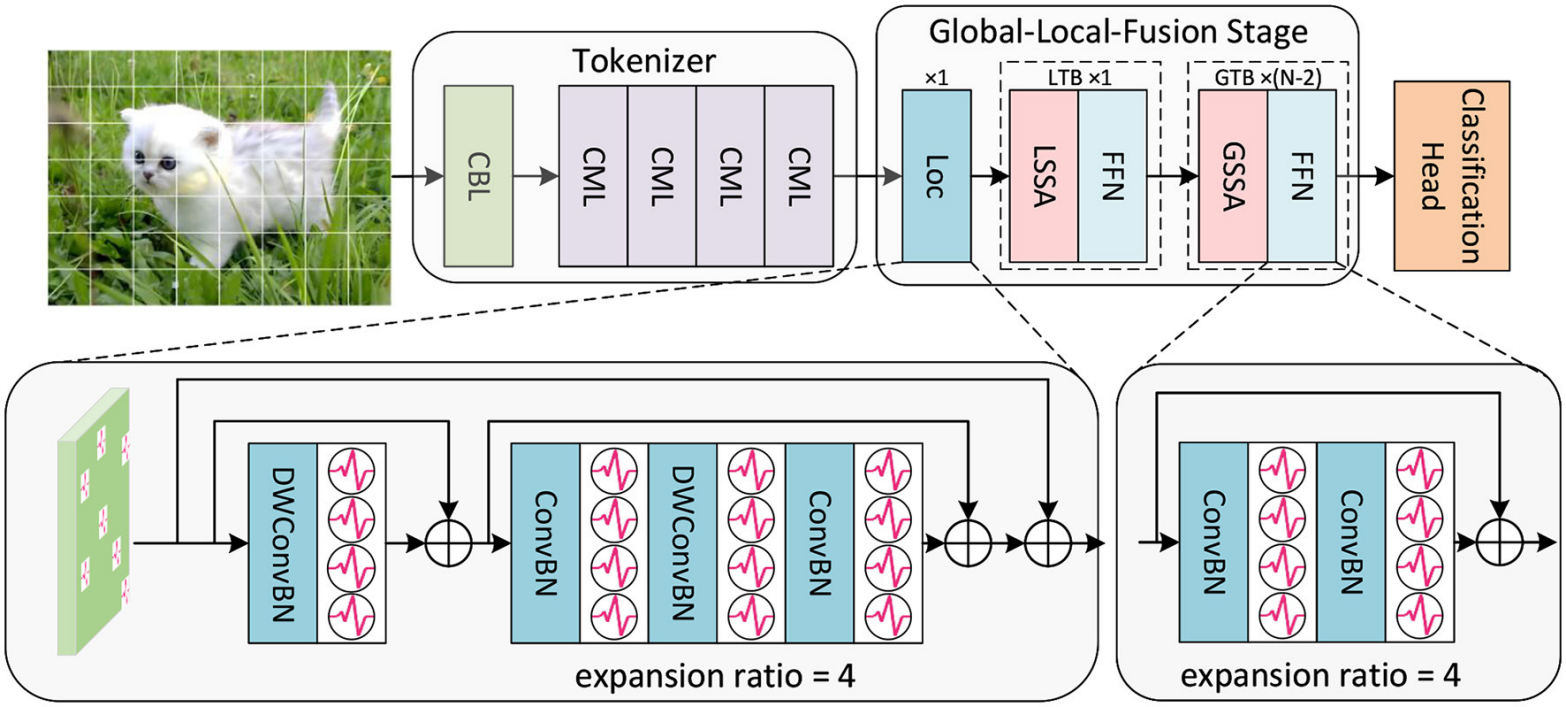
The overall framework of SGLFormer, which consists of a Tokenizer module, a Global-Local-Fusion Stage, and a linear classification head. LTB is a local transformer block, and GTB is a global transformer block.

Inspired by the biological neural system, we integrate the convolutional structure and the transformer structure in SNNs to construct the high-performance SGLFormer. The overall framework of SGLFormer is shown in Figure 1. The SGLFormer includes a Tokenizer module, a Global-Local-Fusion Stage, and a linear classification head. The neuron used in SGLFormer is LIF (Leaky Integrate-and-Fire), which is simple but retains biological characteristics. The dynamics of LIF are described as Equations (1–3):


(1) $$\begin{array}{l} H\left[t\right]=V\left[t-1\right]+\frac{1}{\tau }\left(X\left[t\right]-\left(V\left[t-1\right]-V_{reset}\right)\right) \end{array}$$

(2) $$\begin{array}{l} S\left[t\right]=\Theta \left(H\left[t\right]-V_{th}\right) \end{array}$$

(3) $$\begin{array}{l} V\left[t\right]=H\left[t\right]\left(1-S\left[t\right]\right)+V_{reset}S\left[t\right] \end{array}$$

where τ in Equation (1) is the membrane time constant, *X*[*t*] is the input current at time step *t*. *V*_*reset*_ represents the reset potential, *V*_*th*_ represents the spike firing threshold, *H*[*t*] and *V*[*t*] represent the membrane potential before and after firing spike at time step *t*, respectively. Θ(*v*) is the Heaviside step function, if *v* ≥ 0 then Θ(*v*) = 1, means that firing a spike, otherwise Θ(*v*) = 0. *S*[*t*] represents the output of neuron at time step *t*.

Given a 2D image sequence $I\in {\mathbb{R}}^{T\times B\times 3\times H_{input}\times W_{input}}$ (or a neuromorphic event sequence $I\in {\mathbb{R}}^{T\times B\times 2\times H_{input}\times W_{input}}$), *T* is time steps, and *B* is batch size. The Tokenizer module contains 1 CBL and 4 CMLs. CBL is the abbreviation of ConvBN-LIF, and CML is our SNN-optimized Maxpooling to address the problem of inaccurate gradient backpropagation caused by inappropriate Maxpooling in SNNs. The Tokenizer module is used for feature extraction, channel dimension expansion, and patch embedding. The output of the Tokenizer is $X_{Token}\in {\mathbb{R}}^{T\times B\times C\times H\times W}$.

Each Global-Local-Fusion Stage contains *N* blocks. The number of local feature extraction block Loc and local transformer block (LTB) are both 1, and the number of global transformer block (GTB) is *N* − 2. The LTB consists of local spiking self-attention (LSSA) and feedforward network (FFN), and the GTB consists of global spiking self-attention (GSSA) and FFN. The classification head employs STB instead of global average pooling, which facilitates the aggregation of spatial and temporal features. The overall framework of SGLFormer with *N* = 3 is expressed as Equations (4–8):


(4) $$\begin{array}{l} X_{Token}=\mathrm{Tokenizer}\left(I\right),\;I\in {\mathbb{R}}^{T\times B\times 3\times H_{input}\times W_{input}} \end{array}$$

(5) $$\begin{array}{l} X_{Loc}=\mathrm{Loc}\left(X_{Token}\right),\;X_{Token}\in {\mathbb{R}}^{T\times B\times C\times H\times W} \end{array}$$

(6) $$\begin{array}{l} X_{LTB}=\mathrm{FFN}\left(\mathrm{LSSA}\left(X_{Loc}\right)\right),\;X_{Loc}\in {\mathbb{R}}^{T\times B\times C\times H\times W} \end{array}$$

(7) $$\begin{array}{l} X_{GTB}=\mathrm{FFN}\left(\mathrm{GSSA}\left(X_{LTB}\right)\right),\;X_{LTB}\in {\mathbb{R}}^{T\times B\times C\times H\times W} \end{array}$$

(8) $$\begin{array}{l} Y=\mathrm{Classify}\left(X_{GTB}\right),\;X_{GTB}\in {\mathbb{R}}^{T\times B\times C\times H\times W},Y\in {\mathbb{R}}^{C} \end{array}$$

In the above equations, *H*_*input*_, *W*_*input*_, *H*, and *W* are the height of the input data, the width of the input data, the height of the intermediate feature maps, and the width of the intermediate feature maps, respectively, and *C* is the number of channels and the embedding dimension of the SGLFormer. Classify(·) denotes the classification head operation.

### 2.2 Global-Local-Fusion Stage

The primary visual cortex in the brain mainly extract local information, while the higher-level brain regions focus on abstract high-level information. Based on this, we propose a Global-Local-Fusion Stage that integrates local feature extraction first and then global self-attention. Loc, LTB, and GTB together constitute the Global-Local-Fusion Stage. Loc contains convolution and depthwise convolution, and the expansion ratio controls the number of feature map channels. For example, if the expansion ratio is 4, the convolution layer in Loc will first lift the number of channels to *C* × 4, and then drop back to the original number of channels *C*. The FFN in LTB and GTB, like that in the Loc, controls the number of channels by the expansion ratio. GSSA in GTB is equal to SSA in Spikformer, which is global spiking self-attention. With the input feature map $X_{input}^{G}\in {\mathbb{R}}^{T\times B\times C\times H\times W}$, the computation of GSSA (SSA) is as Equations (9–12):


(9) $$\begin{array}{l} Q^{\prime }=\text{CB}{\text{L}}_{Q}\left(X_{input}^{G}\right),\;K^{\prime }=\text{CB}{\text{L}}_{K}\left(X_{input}^{G}\right),\;V^{\prime }=\text{CB}{\text{L}}_{V}\left(X_{input}^{G}\right) \end{array}$$

(10) $$\begin{array}{l} Q,K,V=\text{Reshape}\left(Q^{\prime },K^{\prime },V^{\prime }\right),\;Q^{\prime },K^{\prime },V^{\prime }\in {\mathbb{R}}^{T\times B\times C\times H\times W} \end{array}$$

(11) $$\begin{array}{l} X_{GSSA}^{\prime }=\text{Reshape}\left(\text{LIF}\left(QK^{T}V\times f\right)\right),\;Q,K,V\in {\mathbb{R}}^{T\times B\times C\times N} \end{array}$$

(12) $$\begin{array}{l} X_{GSSA}=\text{CBL}\left(X_{GSSA}^{\prime }\right),\;X_{GSSA},X_{GSSA}^{\prime }\in {\mathbb{R}}^{T\times B\times C\times H\times W} \end{array}$$

where CBL(·) is ConvBN-LIF, in which the convolution kernel size is 1 × 1 and stride is 1, and *f* is scaling factor, *N* = *H* × *W* is the number of tokens.

**Figure 2:**
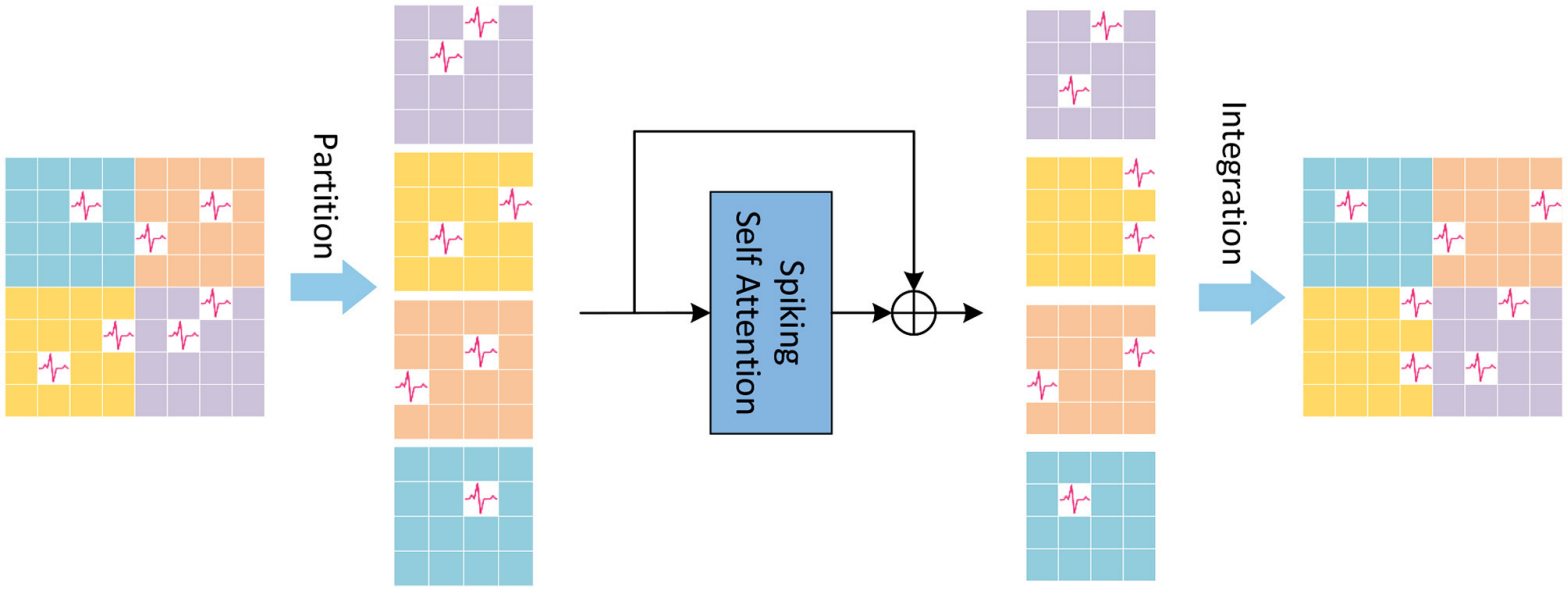
The Local Spiking Self Attention (LSSA), which first divides the feature map into four equally sized parts, and integrates it into the original size after spiking self-attention calculation performed inside the divided feature map.

LSSA in LTB is local spiking self-attention, of which the schematic diagram is shown in Figure 2. In the LSSA block, the input feature map $X_{input}^{L}\in {\mathbb{R}}^{T\times B\times C\times H\times W}$ is first partitioned into 2 × 2 small feature maps, and the height and width of each small feature map are $\frac{H}{2}$ and $\frac{W}{2}$, respectively. After that, spiking self-attention is calculated in each partitioned small feature map. The parameters of spiking self-attention for each partitioned small feature map are shared to reduce computational cost. The small feature maps are then restored to original size for integration. The computation of LSSA is as Equations (13–15):


(13) $$\begin{array}{l} X_{Partition,i}=\text{Partition}\left(X_{input}^{L}\right),\;i\in \left[1,4\right] \end{array}$$

(14) $$\begin{array}{l} X_{SSA,i}=\text{SSA}\left(X_{Partition,i}\right),\;X_{Partition,i},X_{SSA,i}\in {\mathbb{R}}^{T\times B\times C\times \frac{H}{2}\times \frac{W}{2}} \end{array}$$

(15) $$\begin{array}{l} X_{Integration}=\text{Integration}\left(X_{SSA,1},X_{SSA,2},X_{SSA,3},X_{SSA,4}\right) \end{array}$$

where $X_{Integration}\in {\mathbb{R}}^{T\times B\times C\times H\times W}$ is the output feature map of LSSA.

### 2.3 SNN-optimized Maxpooling: CML

We observe that the existing downsampling in SNNs yields inaccurate backpropagation gradients, which can hamper the development and performance improvement of SNNs. We address the problem of inaccurate gradient backpropagation in SNNs by CML.

#### 2.3.1 Inaccurate gradient backpropagation

**Figure 3:**
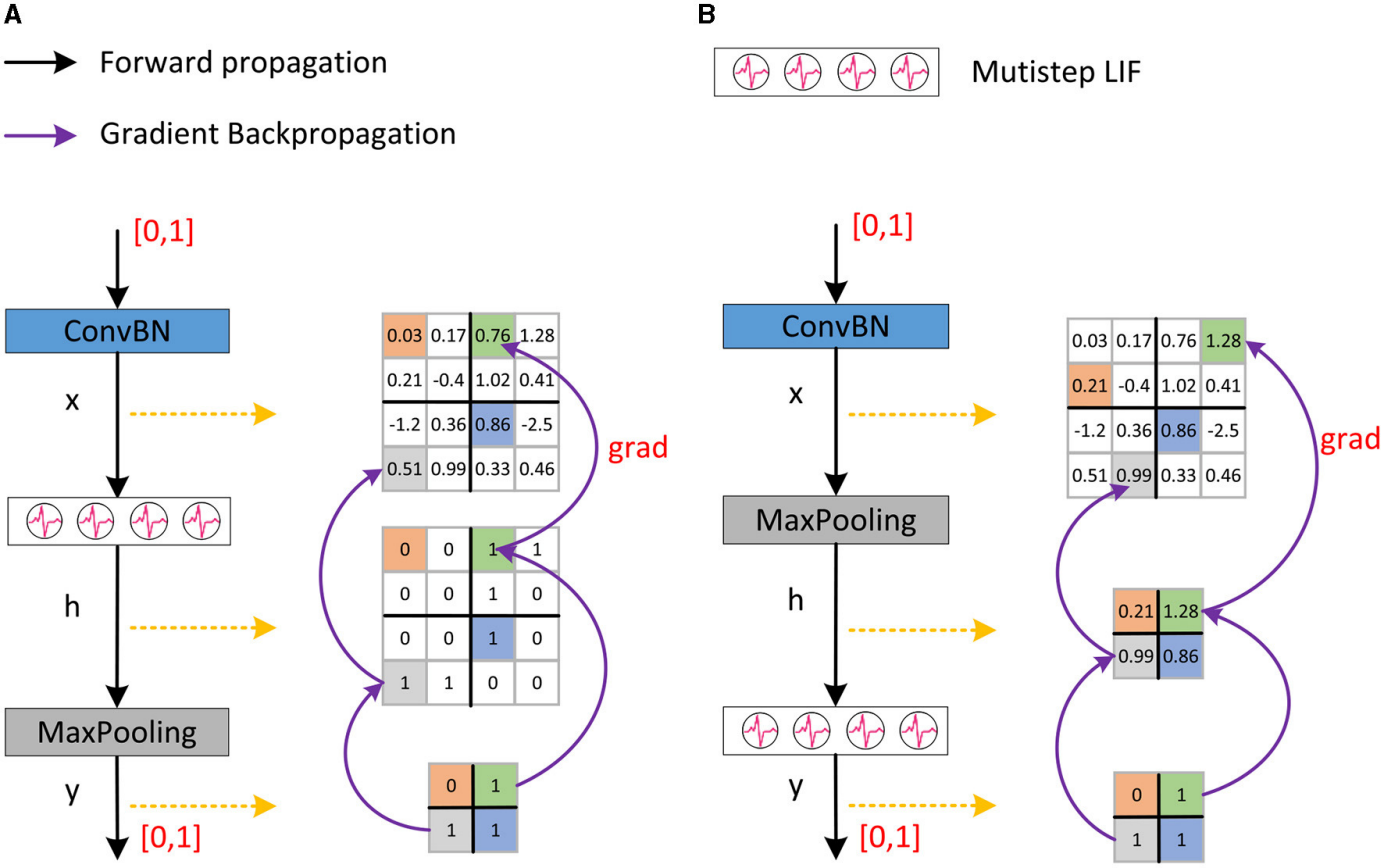
The downsampling method in Spikformer and our proposed SNN-optimized Maxpooling. **(A)** Shows the downsampling method in Spikformer which has an inaccurate gradient backpropagation issue. **(B)** Shows our proposed SNN-optimized Maxpooling (CML) with accurate gradient backpropagation.

SNNs typically employ the network module shown in Figure 3A, i.e., ConvBN-LIF-Maxpooling (CLM), which gives rise to the problem of inaccurate gradient backpropagation. ConvBN represents the combination of convolution and batch normalization. Following ConvBN are the spiking neurons, which receive the resultant current, accumulate the membrane potential across time, and fire a spike when the membrane potential exceeds the threshold. Maxpooling is performed after spiking neurons for downsampling. The output of ConvBN, spiking neurons, and Maxpooling layers, are feature maps *x* ∈ ℝ^*m* × *n*^, *h* ∈ ℝ^*m* × *n*^, and $y\in {\mathbb{R}}^{\frac{m}{s}\times \frac{n}{s}}$ respectively, where *s* is the pooling stride.

Given the loss function *L* and the backpropagation gradient $\frac{\partial L}{\partial y_{ij}}$ after downsampling, the gradient at the feature map *x* is as Equation (16):


(16) $$\begin{array}{l} \frac{\partial L}{\partial x_{uv}}=\overset{\frac{m}{s}}{\underset{i=0}{\sum }}\overset{\frac{n}{s}}{\underset{j=0}{\sum }}\frac{\partial L}{\partial y_{ij}}\frac{\partial y_{ij}}{\partial h_{uv}}\frac{\partial h_{uv}}{\partial x_{uv}} \end{array}$$

The backpropagation gradient of Maxpooling is as Equation (17):


(17) $$\begin{array}{l} \frac{\partial y_{ij}}{\partial h_{uv}}=\{\begin{array}{ll} 1, & h_{uv}=\max\left(h_{i\times s+k,j\times s+r}\right)\\ 0, & others \end{array} \end{array}$$

where *k, r* ∈ [0, *s*). The backpropagation gradient of the LIF neuron is:


(18) $$\begin{array}{l} \frac{\partial h_{uv}}{\partial x_{uv}}=\frac{\partial S\left[t\right]}{\partial X\left[t\right]}=\frac{1}{\tau }\times \Theta^{\prime }\left(H\left[t\right]-V_{th}\right) \end{array}$$

The Θ′(·) in Equation (18) is surrogate gradient. As a result, the backpropagation gradient on feature map *x* is:


(19) $$\begin{array}{l} \frac{\partial L}{\partial x_{uv}}=\{\begin{array}{ll} \frac{1}{\tau }\frac{\partial L}{\partial y_{ij}}\times \Theta^{\prime }\left(H\left[t\right]-V_{th}\right), & h_{uv}=\max\left(h_{i\times s+k,j\times s+r}\right)\\ 0, & others \end{array} \end{array}$$

According to Equation (19), the gradient exists in the position of the maximal element in the feature map *h*. However, the outputs of LIF neurons are spikes, that is, the corresponding value is 1 or 0. In practice, deep learning frameworks such as Pytorch (Paszke et al., 2019) will automatically select the first element with a value of 1 as the maximum value in feature map *h* during backpropagation. There is no gradient in the position of other elements with a value of 1, which causes inaccurate gradient backpropagation. To sum up, after downsampling, when conducting backpropagation in the network module shown in Figure 3A, the element with a gradient in the feature map *x* is not necessarily the element with most feature information, which is the problem of inaccurate gradient backpropagation.

#### 2.3.2 Optimized Maxpooling

In binary neural networks, XNOR-net (Rastegari et al., 2016) places the pooling behind the convolution and BN before the activation function to address the above problem, forming a BN-BinActiv-BinConv-Pool structure. Datta et al. (2022) proposed a similar structure as XNOR-net in SNNs. Here, we improve the downsampling by placing the LIF neuron layer after the Maxpooling layer, as shown in Figure 3B. The improved structure is ConvBN-Maxpooling-LIF, named CML, which addresses the inaccurate gradient backpropagation. The output of ConvBN, Maxpooling, and spiking neuron layer are feature map *x* ∈ ℝ^*m* × *n*^, $h\in {\mathbb{R}}^{\frac{m}{s}\times \frac{n}{s}}$, and $y\in {\mathbb{R}}^{\frac{m}{s}\times \frac{n}{s}}$ respectively, where *s* is the pooling stride. The backpropagation gradient $\frac{\partial L}{\partial y_{ij}}$ after the LIF neuron is known, then the gradient at the feature map *x* is as Equation (20):


(20) $$\begin{array}{l} \frac{\partial L}{\partial x_{uv}}=\overset{\frac{m}{s}}{\underset{i=0}{\sum }}\overset{\frac{n}{s}}{\underset{j=0}{\sum }}\frac{\partial L}{\partial y_{ij}}\frac{\partial y_{ij}}{\partial h_{ij}}\frac{\partial h_{ij}}{\partial x_{uv}} \end{array}$$

The backpropagation gradient of Maxpooling is as Equation (21):


(21) $$\begin{array}{l} \frac{\partial h_{ij}}{\partial x_{uv}}=\{\begin{array}{ll} 1, & x_{uv}=\max\left(x_{i\times s+k,j\times s+r}\right)\\ 0, & others \end{array} \end{array}$$

where *k, r* ∈ [0, *s*). The backpropagation gradient of the LIF neuron is as Equation (22):


(22) $$\begin{array}{l} \frac{\partial y_{ij}}{\partial h_{ij}}=\frac{\partial S\left[t\right]}{\partial X\left[t\right]}=\frac{1}{\tau }\times \Theta^{\prime }\left(H\left[t\right]-V_{th}\right) \end{array}$$

As a result, the backpropagation gradient on feature map *x* is as follows:


(23) $$\begin{array}{l} \frac{\partial L}{\partial x_{uv}}=\{\begin{array}{ll} \frac{1}{\tau }\frac{\partial L}{\partial y_{ij}}\times \Theta^{\prime }\left(H\left[t\right]-V_{th}\right) & ,x_{uv}=\max\left(x_{i\times s+k,j\times s+r}\right)\\ 0 & ,others \end{array} \end{array}$$

According to Equation (23), the maximum element in feature map *h* corresponds to the maximum element in feature map *x*. Thus, after downsampling, when conducting backpropagation in the network structure shown in Figure 3B, the element with a gradient in feature map *x* is the element with the most feature information, addressing the problem inaccurate of gradient backpropagation. In addition, the computational cost of CML with *s* = 2 on LIF neurons is only one-quarter of the downsampling process in Figure 3A, which is more in line with the low energy consumption characteristic of biological nervous systems and SNNs.

### 2.4 Classification head

**Figure 4:**
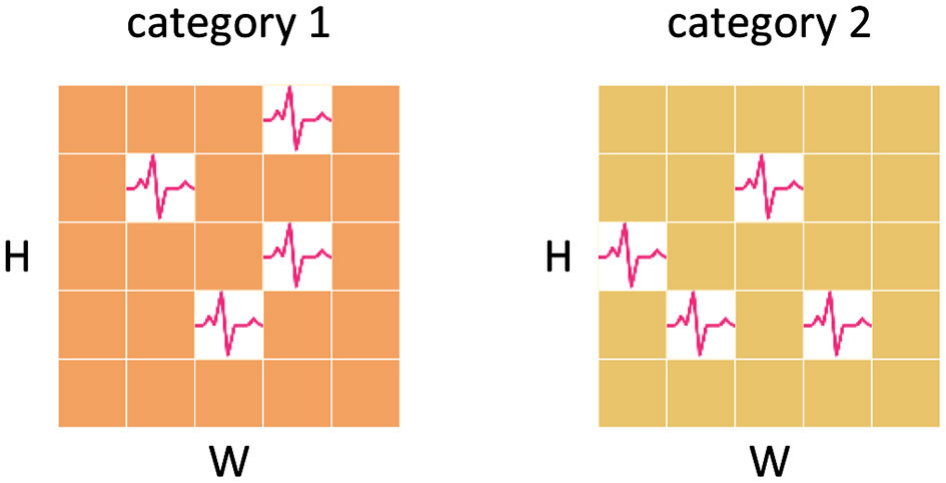
Information loss caused by averaging. The feature maps of both categories have the same number of spikes and the feature representations obtained by averaging are consistent, which results in the loss of temporal and spatial information.

In the typical classification head of SNNs, the standard approach involves averaging the feature maps generated by the backbone across the spatial and temporal domains as the spatio-temporal feature representation. This method ignores the non-uniformity of spatial and temporal spike distribution. It fails to account for the possibility that feature maps of different classes, may exhibit distinct spike distributions although with the same number of spikes, as shown in Figure 4. This oversight can lead to a loss of valuable spatial and temporal information, thus derogating the performance of SNNs.

**Figure 5:**
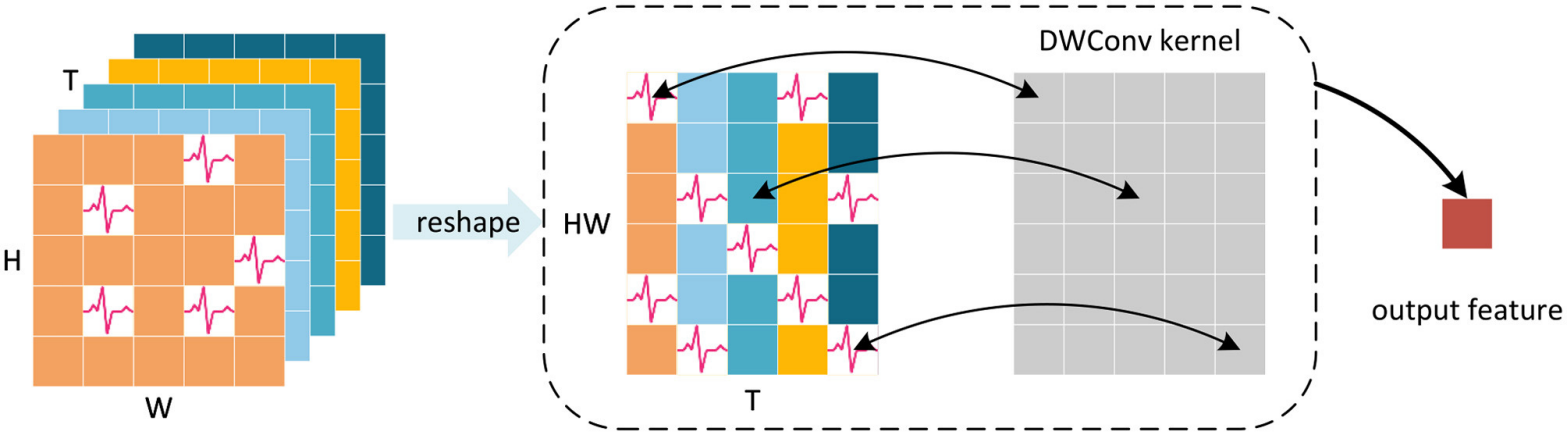
Schematic diagram of STB for single channel feature map. Depthwise convolution (DWConv) uses convolution kernels with the same size as the reshaped feature map to aggregate spatial and temporal information, avoiding the information loss caused by averaging.

To address this limitation, we have developed a new classification head to preserve more crucial spatial and temporal information. We first perform a reshape operation on the feature map generated by the Global-Local-Fusion Stage, and then use STB instead of global average pooling in spatial and temporal domains to extract spike distribution information, as shown in Figure 5. The convolutional kernel size of STB is consistent with the size of the feature map, and it aggregates spatial and temporal information to generate a 1 × 1 feature representation. The classification head works with input feature map $X_{input}^{Cla}\in {\mathbb{R}}^{T\times B\times C\times H\times W}$ as Equations (24–26):


(24) $$\begin{array}{l} X_{Reshape}=\text{Reshape}\left(X_{input}^{Cla}\right) \end{array}$$

(25) $$\begin{array}{l} X_{STB}=\text{STB}\left(X_{Reshape}\right),\;X_{Reshape}\in {\mathbb{R}}^{B\times C\times HW\times T} \end{array}$$

(26) $$\begin{array}{l} Y=\text{Linear}\left(\text{BN}\left(X_{STB}\right)\right),\;X_{STB}\in {\mathbb{R}}^{B\times C\times 1\times 1} \end{array}$$

In the above equation, *Y* ∈ ℝ^*C*^is the output of SGLFormer. STB uses a large convolutional kernel, but it is depthwise convolution (DWConv), which ensures that the addition of parameters remains minimal. Moreover, there is no nonlinear activation function in the classification head, so the STB, BN, and Linear can be fused into a linear convolutional layer during the inference stage.

Suppose a batch in the model training phase contains *B* samples, *x*_1_, *x*_2_, …, *x*_*B*_. For the *i*-th sample, STB without bias term can be written as follows:


(27) $$\begin{array}{l} \begin{array}{l} x_{i,c}=\overset{H*W}{\underset{j=1}{\sum }}\overset{T}{\underset{t=1}{\sum }}w_{j,t}x_{i,c,j,t}^{re}\\ =w_{STB}*x_{i,c}^{re} \end{array} \end{array}$$

where *x*_*i,c*_ in Equation (27) is the feature map of the *i*-th sample and the *c*-th channel, $x_{i,c}^{re}\in {\mathbb{R}}^{HWT\times 1}$ is the feature map of the *c*-th channel after reshape, $w_{STB}\in {\mathbb{R}}^{1\times HWT}$ is the weight of the STB in *c*-th channel, and the symbol * denotes matrix multiplication.

The BN operation on the *c*-th channel can be written as Equation (28):


(28) $$\begin{array}{l} \begin{array}{l} {\hat{x}}_{i,c}=\gamma_{c}\frac{x_{i,c}-\mu_{c}}{\sqrt{\sigma_{c}^{2}+\epsilon }}+\beta_{c}\\ =\frac{\gamma_{c}x_{i,c}}{\sqrt{\sigma_{c}^{2}+\epsilon }}+\beta_{c}-\frac{\gamma_{c}\mu_{c}}{\sqrt{\sigma_{c}^{2}+\epsilon }} \end{array} \end{array}$$

where γ_*c*_, β_*c*_, μ_*c*_, and $\sigma_{c}^{2}$ are the scaling coefficient, translation coefficient, mean and variance of the *c*-th channel, respectively. For all channels, the BN operation can be written in the form of matrix multiplication as follows:


(29) $$\begin{array}{l} \left[\begin{array}{l} {\hat{x}}_{i,1}\\ {\hat{x}}_{i,2}\\ \vdots \\ {\hat{x}}_{i,C-1}\\ {\hat{x}}_{i,C} \end{array}\right]=\left[\begin{array}{lllll} \frac{\gamma_{1}}{\sqrt{\sigma_{1}^{2}+\epsilon }} & 0 & \cdots & 0 & 0\\ 0 & \frac{\gamma_{2}}{\sqrt{\sigma_{2}^{2}+\epsilon }} & \vdots \\ \vdots & \ddots & \frac{\gamma_{C-1}}{\sqrt{\sigma_{C-1}^{2}+\epsilon }} & 0\\ 0 & 0 & \cdots & 0 & \frac{\gamma_{C}}{\sqrt{\sigma_{C}^{2}+\epsilon }} \end{array}\right]\\ \cdot \left[\begin{array}{l} x_{i,1}\\ x_{i,2}\\ \vdots \\ x_{i,C-1}\\ x_{i,C} \end{array}\right]+\left[\begin{array}{l} \beta_{1}-\gamma_{1}\frac{\mu_{1}}{\sqrt{\sigma_{1}^{2}+\epsilon }}\\ \beta_{2}-\gamma_{2}\frac{\mu_{2}}{\sqrt{\sigma_{2}^{2}+\epsilon }}\\ \vdots \\ \beta_{C-1}-\gamma_{C-1}\frac{\mu_{C-1}}{\sqrt[{4}]{\sigma_{C-1}^{2}+\epsilon }}\\ \beta_{C}-\gamma_{C}\frac{\mu_{C}}{\sqrt{\sigma_{C}^{2}+\epsilon }} \end{array}\right] \end{array}$$

Equation (29) can be rewritten as Equation (30):


(30) $$\begin{array}{l} {\hat{x}}_{i}=W_{BN}*x_{i}+b_{BN} \end{array}$$

where diagonal matrix $W_{BN}\in {\mathbb{R}}^{C\times C}$ and $b_{BN}\in {\mathbb{R}}^{C}$ are the parameters of BN. Then STB, BN, and Linear layers of the classification head fused into one convolutional layer which can be written as follows:


(31) $$\begin{array}{l} \begin{array}{l} y_{i}=W_{Linear}\left(W_{BN}\left(W_{STB}*x_{i}^{re}\right)+b_{BN}\right)\\ =W_{Linear}W_{BN}W_{STB}*x_{i}^{re}+W_{Linear}b_{BN}\\ =W_{fusion}*x_{i}^{re}+b_{fusion} \end{array} \end{array}$$

In the Equation (31), $W_{Linear}\in {\mathbb{R}}^{C\times C}$ is the weight of Linear layer, $W_{STB}\in {\mathbb{R}}^{C\times 1\times HWT}$ is the weight of STB, and $x_{i}^{re}\in {\mathbb{R}}^{C\times HWT\times 1}$ is the feature map after reshape.

### 2.5 Synaptic operations and energy consumption

We first calculate the number of the synaptic operations (SOP) of neurons as Equation (32):


(32) $$\begin{array}{l} SOP^{i}=fr\times T\times FLOP^{i} \end{array}$$

where *fr* is the firing rate of a layer and *T* is the time steps. *FLOP*^*i*^ refers to floating point operations of layer *i*, which is the number of multiply-and-accumulate (MAC) operations. And *SOP*^*i*^ is the number of spike-based accumulate (AC) operations.

Assuming the MAC and AC operations are performed on the 45 nm hardware (Horowitz, 2014), *E*_*MAC*_ = 4.6*pJ* and *E*_*AC*_ = 0.9*pJ*. We estimate the energy consumption of SGLFormer according to Zhou C. et al. (2023). The energy consumption of SGLFormer can be calculated as follows:


(33) $$\begin{array}{l} E_{SGLFormer}=E_{AC}\times \left(\overset{N}{\underset{i=2}{\sum }}SOP_{Conv}^{i}+\overset{M}{\underset{j=1}{\sum }}SOP_{SSA}^{j}\right)\\ +E_{MAC}\times \left(FLOP_{Conv}^{1}\right) \end{array}$$

where $FLOP_{Conv}^{1}$ represents the first layer encoding input into spike-form, *SOP*_*Conv*_ represents the SOP of the a convolution layer, and *SOP*_*SSA*_ represents the SOP of a LSSA or GSSA. *N* is the number of convolution layers and *M* is the number of LSSA and GSSA layers.

## 3 Results

In this section, we evaluate the performance of SGLFormer on static datasets CIFAR10/CIFAR100 (Krizhevsky, 2009), ImageNet (Deng et al., 2009), as well as neuromorphic datasets CIFAR10-DVS (Li et al., 2017), and DVS128-Gesture (Amir et al., 2017). The deep learning frameworks used to implement the experiment are PyTorch (Paszke et al., 2019), Timm (Wightman, 2019), and SpikingJelly (Fang et al., 2023). We train SGLFormer from scratch and compare it with existing SNNs to show that SGLFormer achieves SOTA performance. Most of the hyperparameters in training follow Spikformer (Zhou Z. et al., 2023). In addition, we conduct an ablation study on CIFAR100 and calculate the energy consumption of the SGLFormer.

### 3.1 Results on static datasets

**ImageNet** training set contains more than 1.2 million images and the validation set contains 50,000 images for testing, where all the images belong to 1,000 categories. In both the training phase and the testing phase, the input size of the network is 224 × 224. The number of training epochs is set to 200, with a cosine-decay learning rate whose initial value is set empirically to 0.0012. We adopt a batch size of 512, distributed across 8 Nvidia V100 GPUs. Unlike the CIFAR datasets, SGLFormer on ImageNet does not use STB, and the Mixup data augmentation technique is also excluded. The experimental results on the ImageNet dataset are shown in Table 1. The results show that SGLFormer comprehensively outperforms existing non-transformer networks such as SEW ResNet, MS-ResNet, and Att MS-ResNet. The performance of SGLFormer-8-384 is 78.50%, surpassing the highest performance of SEW ResNet 69.26% by 9.24%, and surpassing the highest performance of MS-ResNet 76.02% by 2.48% and Att MS-ResNet 77.08% by 1.42%.

**Table 1:** Experimental results on ImageNet.

**Dataset**	**Methods**	**Architecture**	**Param (M)**	**Time step**	**Top-1 Acc (%)**
ImageNet	Hybrid training (Rathi et al., 2020)	ResNet-34	21.79	250	61.48
	STBP-tdBN (Zheng et al., 2021)	Spiking-ResNet-34	21.79	6	63.72
	TET (Deng et al., 2021)	Spiking-ResNet-34	21.79	6	64.79
		SEW-ResNet-34	21.79	4	68.00
	Spiking ResNet (Hu et al., 2023b)	ResNet-34	21.79	350	71.61
		ResNet-50	25.56	350	72.75
	SEW ResNet (Fang et al., 2021a)	SEW-ResNet-34	21.79	4	67.04
		SEW-ResNet-50	25.56	4	67.78
		SEW-ResNet-101	44.55	4	68.76
		SEW-ResNet-152	60.19	4	69.26
	MS-ResNet (Hu et al., 2021)	MS-ResNet-18	11.69	6	63.10
		MS-ResNet-34	21.80	6	69.42
		MS-ResNet-104	77.28	5	76.02
	Att MS-ResNet (Yao et al., 2023b)	Att-MS-ResNet-18	11.87	1	63.97
		Att-MS-ResNet-34	22.12	1	69.15
		Att-MS-ResNet-104	78.37	4	77.08
	ANN (Zhou Z. et al., 2023)	Transformer-8-512	29.68	-	80.80
	Spikformer (Zhou Z. et al., 2023)	Spikformer-8-384	16.81	4	70.24
		Spikformer-8-512	29.68	4	73.38
		Spikformer-8-768	66.34	4	74.81
	Spikingformer (Zhou C. et al., 2023)	Spikingformer-8-384	16.81	4	72.45
		Spikingformer-8-512	29.68	4	74.79
		Spikingformer-8-768	66.34	4	75.85
	S-Transformer (Yao et al., 2023a)	S-Transformer-8-384	16.81	4	72.28
		S-Transformer-8-512	29.68	1	71.68
		S-Transformer-8-512	29.68	4	74.57
		S-Transformer-8-768	66.34	4	77.07
	**SGLFormer**	SGLFormer-8-384	16.25	4	**79.44**
		SGLFormer-8-512	28.67	4	**82.28**
		SGLFormer-8-512^*^	28.67	4	**81.93**
		SGLFormer-8-768^*^	64.02	4	**83.73**

SGLFormer-8-384 indicates that the total number of Loc, LTB, and GTB in the Global-Local-Fusion Stage of SGLFormer is 8, and the embedding dimension of SGLFormer is 384. Note that 83.73% of SGLFormer-8-768^*^ achieves the SOTA performance on ImageNet in directly trained SNN models. The bold method represents our model, and accuracy. ^*^indicates that the last layer of the Tokenizer is the CBL block and other layers are CML blocks.

Compared with transformer-based SNNs, SGLFormer has fewer parameters and higher performance under the same embedding dimension. The accuracy of SGLFormer-8-384 is 79.44%, which is 9.20% higher than that of Spikformer-8-384, 6.99% higher than that of Spikingformer-8-384, and 7.16% higher than that of S-Transformer-8-384. The accuracy of SGLFormer-8-512 is 82.28%, which is 8.90% higher than that of Spikformer-8-512, 7.49% higher than that of Spikingformer-8-512, and 7.71% higher than that of S-Transformer-8-512. Moreover, SGLFormer-8-512 outperforms Transformer-8-512 by 1.48% in accuracy, which is the ANN counterpart of Spikformer-8-512. The Tokenizer module of SGLFormer is composed of 1 CBL block and 4 CML blocks. The first block is CBL if not specified. To speed up the training phase of the model, we swap the positions of the CBL block and CML blocks, that is, the last layer of the Tokenizer is the CBL block. The resulting model is named SGLFormer-8-512^*^, and its performance is reduced by 0.35% compared with the original model. The highest performance model in SGLFormer is SGLFormer-8-768^*^, with an accuracy of 83.73%, which is 8.92% higher than that of Spikformer-8-768, 7.88% higher than that of Spikingformer-8-768, and 6.66% higher than that of S-Transformer-8-768.

**CIFAR10/CIFAR100** each has a total of 60,000 images, including 50,000 in the training set and 10,000 in the testing set. The input size is the same as the image resolution, which is 32 × 32. CIFAR10 and CIFAR100 have 10 and 100 categories, respectively. The batch size is set to 64, and the number of training epochs is set to 410, with a cosine-decay learning rate whose initial value is set empirically to 0.001. AdamW is used as the optimizer. The static image input in SGLFormer needs to be repeated *T* times in the time dimension. Data augmentation techniques such as Mixup, Random erase, and Horizontal flip are used in the training process. After the calculation by the Tokenizer module, the input image is divided into 8 × 8 patches, and the size of each patch is 4 × 4.

The experimental results on CIFAR10 and CIFAR100 datasets are shown in Table 2. SGLFormer has fewer parameters and outperforms all other models. On the CIFAR10 dataset, the SGLFormer-4-384 model with 8.85M parameters achieves a performance of 96.76%. The number of parameters of SGLFormer-4-384 is only 50.46 and 70.07% of STBP and STBP-tdBN, respectively, while outperforming them by margins of 6.93 and 3.84%. Compared with Spikformer-4-384 and Spikingformer-4-384, our SGLFormer-4-384 reduces the number of parameters by 0.47 M, but improves the performance by 1.25 and 0.95%, respectively. In addition, SGLFormer-4-384 has 1.43M fewer parameters than S-Transformer-2-512 but has 1.16% higher performance.

**Table 2:** Experimental results on CIFAR10, CIFAR100.

**Dataset**	**Methods**	**Architecture**	**Param (M)**	**Time step**	**Top-1 Acc (%)**
CIFAR10	STBP (Wu et al., 2018)	CIFARNet	17.54	12	89.83
	STBP-tdBN (Zheng et al., 2021)	ResNet-19	12.63	4	92.92
	Spikformer (Zhou Z. et al., 2023)	Spikformer-2-384	5.76	4	94.80
		Spikformer-4-384	9.32	4	95.51
	Spikingformer (Zhou C. et al., 2023)	Spikingformer-2-384	5.76	4	95.22
		Spikingformer-4-384	9.32	4	95.81
	S-Transformer (Yao et al., 2023a)	S-Transformer-2-512	10.28	4	95.60
	**SGLFormer**	SGLFormer-4-384	8.85	4	**96.76**
CIFAR100	STBP-tdBN (Zheng et al., 2021)	ResNet-19	12.63	4	70.86
	TET (Deng et al., 2021)	ResNet-19	12.63	4	74.47
	Spikformer (Zhou Z. et al., 2023)	Spikformer-2-384	5.76	4	76.95
		Spikformer-4-384	9.32	4	78.21
	Spikingformer (Zhou C. et al., 2023)	Spikingformer-2-384	5.76	4	78.34
		Spikingformer-4-384	9.32	4	79.21
	S-Transformer (Yao et al., 2023a)	S-Transformer-2-512	10.28	4	78.40
	**SGLFormer**	SGLFormer-4-384	8.88	4	**82.26**

The bold method represents our model, and accuracy.

On the CIFAR100 dataset, due to the increase in the number of categories, the number of linear layer parameters of the classification head also increases. Hence, the number of parameters of SGLFormer-4-384 is 8.88M, which is 70.31% of the number of parameters of STBP-tdBN and TET, while the performance is improved by 11.4 and 7.79%, respectively. Compared to other models like Spikformer-4-384, Spikingformer-4-384, and S-Transformer-2-512, our SGLFormer-4-384 demonstrates a reduction in parameters by 0.44, 0.44, and 1.40 M, respectively, while simultaneously achieving performance increments of 4.05, 3.05, and 3.86%.

### 3.2 Results on DVS datasets

**Figure 6:**
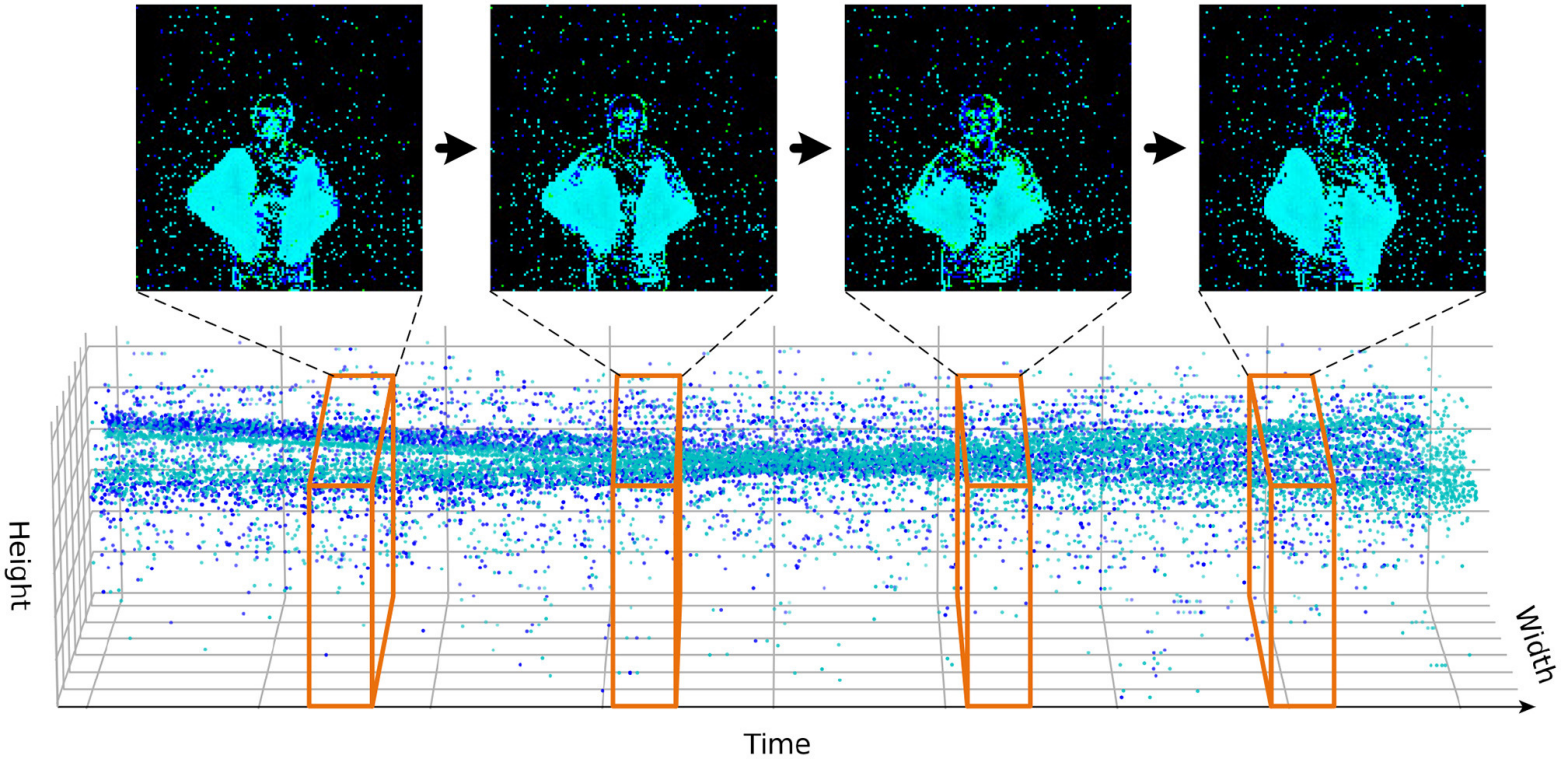
In the DVS data processing scheme, a fixed number of event streams are integrated into frames.

**CIFAR10-DVS** is a neuromorphic dataset converted from the static image dataset. It contains 9,000 training samples and 1,000 testing samples, belonging to 10 categories, and the resolution of the dataset is 128 × 128. We first integrate the events stream into frames with the method of Fang et al. (2021b), as shown in Figure 6. The batch size is 16, the number of time steps of the spiking neuron is 10 or 16, and the number of epochs is 106, which is the same as Spikformer. The learning rate is set empirically to 0.005 and decayed with a cosine schedule.

The experimental results on the CIFAR10-DVS dataset are shown in Table 3. Due to the smaller amount of DVS data compared to static datasets, the embedding dimension used in the experiment is 256, and the expansion ratio and number of blocks in the Global-Local-Fusion Stage are set to 1 and 3 respectively, which means the model is SGLFormer-3-256. SGLFormer-3-256 achieves 82.6% top-1 accuracy with 16 time steps and 82.9% accuracy with 10 time steps, which significantly outperforms Spikformer by 4.0 and 1.7%, outperforms Spikingformer by 3.0 and 1.3%, and outperforms STSFormer by 3.9 and 2.7% respectively. SGLFormer-3-256 outperforms S-Transformer-2-256 by 2.6% with the same time steps.

**Table 3:** Experimental results on CIFAR10-DVS and DVS128-Gesture.

**Dataset**	**Methods**	**Architecture**	**Param (M)**	**Time step**	**Top-1 Acc (%)**
CIFAR10-DVS	Spikformer (Zhou Z. et al., 2023)	Spikformer-2-256	2.57	10	78.9
		Spikformer-2-256	2.57	16	80.9
	Spikingformer (Zhou C. et al., 2023)	Spikingformer-2-256	2.57	10	79.9
		Spikingformer-2-256	2.57	16	81.3
	S-Transformer (Yao et al., 2023a)	S-Transformer-2-256	2.57	16	80.0
	STSA (Wang et al., 2023)	STSFormer-2-256	1.99	10	79.0
		STSFormer-2-256	1.99	16	79.9
	**SGLFormer**	SGLFormer-3-256	2.48	10	**82.9**
		SGLFormer-3-256	2.58	16	**82.6**
DVS128-Gesture	Spikformer (Zhou Z. et al., 2023)	Spikformer-2-256	2.57	10	96.9
		Spikformer-2-256	2.57	16	98.3
	Spikingformer (Zhou C. et al., 2023)	Spikingformer-2-256	2.57	10	96.2
		Spikingformer-2-256	2.57	16	98.3
	S-Transformer (Yao et al., 2023a)	S-Transformer-2-256	2.57	16	99.3
	STSA (Wang et al., 2023)	STSFormer-2-256	1.99	10	97.3
		STSFormer-2-256	1.99	16	98.7
	**SGLFormer**	SGLFormer-3-256	2.08	10	**97.2**
		SGLFormer-3-256	2.17	16	**98.6**

The bold method represents our model, and accuracy.

**DVS128-Gesture** is a neuromorphic dataset for gesture recognition, which contains 11 different classes of gestures collected from 29 individuals under three different illumination conditions. The resolution of DVS128-Gesture and the hyperparameter setting of SGLFormer in classification tasks are consistent with CIFAR10-DVS, except that the number of training epochs is 202. The experimental results on the DVS128-Geature dataset are shown in Table 3. The expansion ratio in the Loc and Global-Local-Fusion Stage is set to 1. SGLFormer-3-256 achieves 98.6% top-1 accuracy with 16 time steps and 97.2% accuracy with 10 time steps, which outperforms Spikformer by 0.3% in different time steps, and outperforms Spikingformer by 1.0 and 0.3%, respectively. SGLFormer-3-256 is slightly worse than S-Transformer-2-256 with fewer parameters and achieves almost the same performance as STSFormer.

### 3.3 Ablation study

**Ablation study** is conducted on the CIFAR100 dataset, and the results are shown in Table 4. The basic model of the ablation study is SGLFormer-4-384. For the Loc block, if the GTB is used to replace it, the number of parameters reaches 9.46 M, while the top-1 accuracy decreases by 1.21%; if the Loc block is directly removed, the number of parameters is reduced to 7.68 M, but its top-1 accuracy is reduced by 1.57%. For LSSA, we replace it with GSSA while keeping the total number of parameters unchanged, resulting in a 0.31% decrease in top-1 accuracy. For STB in the classification head, the performance drops by 0.77% when using the original global average pooling (GAP). For CML, we replace it with the same downsampling method CLM (ConvBN-LIF-MaxPooling) as in Spikformer, and the top-1 accuracy decreases by 1.7% with the number of parameters unchanged. The results of the ablation study show that Loc, LSSA, STB, and CML together contribute to the high performance of SGLFormer.

**Table 4:** Ablation study of modules in SGLFormer, the dataset is CIFAR 100.

**Architecture**	**Loc**	**LSSA**	**STB**	**CML**	**Param (M)**	**Top-1 Acc (%)**
SGLFormer-4-384	✓	✓	✓	✓	8.88	82.26
	GTB	✓	✓	✓	9.46	81.05
	×	✓	✓	✓	7.68	80.69
	✓	GSSA	✓	✓	8.88	81.95
	✓	✓	GAP	✓	8.78	81.49
	✓	✓	✓	CLM	8.88	80.56

Furthermore, we conduct an ablation study on each block in GTB, the results are shown in Table 5, where “base” represents the basic settings of the model SGLFormer-4-384, “number” and “position” respectively represent the number and position ablation study for each block in GTB. We mark the position and number of blocks in GTB, for example, “2, 1 × ” indicates that the position of a certain block is the second and the number is 1. For the number ablation study, we keep the position of the blocks constant, and the total number is unchanged at 4. When the number of LTB is 2, the number of parameters is unchanged, and the top-1 accuracy decreases by 0.21%. When the number of Loc is 2, the number of parameters is slightly reduced, but the top-1 accuracy is decreased by 0.86%. This suggests that basic settings are superior to others in terms of the number of blocks. For the position ablation study, we only change the position of the blocks while keeping the number of each block constant, resulting in all models with the same number of parameters as the “base.” When swapping the positions of LTB and GTB, the top-1 accuracy decreases by 0.41%. Then, by swapping the positions of Loc and LTB, the top-1 accuracy decreased by 0.67%. Finally, swapping the positions of Loc and GTB results in a 1.55% decrease in top-1 accuracy. This indicates that the structure of transitioning from shallow local operations to deep global operations is superior to other structures, and shallow local operations can correspond to local feature extraction in the primary visual cortex of the nervous system, while deep global operations can correspond to abstract information extraction in higher-level brain regions.

**Table 5:** Ablation study of blocks in GTB, the dataset is CIFAR 100.

**Architecture**		**Loc**	**LTB**	**GTB**	**Param (M)**	**Top-1 Acc (%)**
SGLFormer-4-384	Base	1, 1×	2, 1×	3, 2×	8.88	82.26
	Number	1, 1×	2, 2×	3, 1×	8.88	82.05
		1, 2×	2, 1×	3, 1×	8.31	81.40
	Position	1, 1×	3, 1×	2, 2×	8.88	81.85
		2, 1×	1, 1×	3, 2×	8.88	81.59
		3, 1×	2, 1×	1, 2×	8.88	80.71

“2, 1 × ” indicates that the position of a certain block is the second and the number is 1.

### 3.4 Energy consumption

**Energy consumption** of SGLFormer is calculated according to Equation (33) on ImageNet, as shown in Table 6. The energy consumption of SGLFormer-8-384 is 13.04 mJ, which is extremely close to that of SEW-ResNet-152 while its performance far exceeds SEW-ResNet-152. Compared with the Spikformer, the performance of SGLFormer-8-384 significantly exceeds the optimal model Spikformer-8-768, and its energy consumption is reduced by 8.44 mJ, which is only 60.71% of the Spikformer. For ANN, we choose Transformer-8-512, which is the ANN counterpart of Spikformer-8-512. The energy consumption of SGLFormer-8-512 is only 54.64% of that of Transformer-8-512, while the energy consumption of SGLFormer-8-512^*^ is further reduced, only 27.73% of that of Transformer-8-512. At the same time, SGLFormer-8-512 and SGLFormer-8-512^*^ have higher performance. The energy consumption of SGLFormer-8-768^*^ is 19.93 mJ, which is 7.22% less than that of Spikformer-8-768. In summary, our SGLFormer has a huge advantage over non-spike ANN in terms of energy consumption, and further reduces energy consumption compared to existing SNNs with the same performance.

**Table 6:** Energy consumption analysis.

**Methods**	**Architecture**	**Param (M)**	**Time step**	**Power (mJ)**
SEW ResNet (Fang et al., 2021a)	SEW-ResNet-152	60.19	4	12.89
ANN (Zhou Z. et al., 2023)	Transformer-8-512	29.68	-	38.34
Spikformer (Zhou Z. et al., 2023)	Spikformer-8-384	16.81	4	7.73
	Spikformer-8-512	29.68	4	11.58
	Spikformer-8-768	66.34	4	21.48
**SGLFormer**	SGLFormer-8-384	16.25	4	13.04
	SGLFormer-8-512	28.67	4	20.95
	SGLFormer-8-512^*^	28.67	4	10.63
	SGLFormer-8-768^*^	64.02	4	19.93

Power is the average theoretical energy consumption of an image inference on ImageNet. ^*^indicates that the last layer of the Tokenizer is the CBL block and other layers are CML blocks.

## 4 Discussion

In this work, we propose a high-performance Spiking Global-Local-Fusion Transformer, named SGLFormer. SGLFormer integrates convolutional structure and transformer structure that processes local information and global information, respectively, to fill the performance gap between SNNs and ANNs. CML is designed as SNN-optimized Maxpooling to address the problem of inaccurate gradient backpropagation caused by inappropriate Maxpooling in SNNs. Furthermore, STB is used to improve the classification head so that it can facilitate efficient aggregation of spatial and temporal features. We evaluate the performance of SGLFormer on both static datasets and DVS datasets. Experimental results show that SGLFormer significantly outperforms existing SOTA methods in directly trained SNNs and closely approaches the performance of SOTA ANNs trained from scratch.

To support more computer vision tasks as a backbone network, SGLFormer can be further improved through the combination of pre-training and fine-tuning. For example, pre-training on the image classification dataset ImageNet and fine-tuning on datasets for other visual tasks. Moreover, considering the deployment of SGLFormer in edge devices, further optimizations could be focused on reducing the number of parameters and simplifying the network structure, without performance degradation.

## Data availability statement

The original contributions presented in the study are included in the article/Supplementary material, further inquiries can be directed to the corresponding authors.

## Author contributions

HZha: Methodology, Writing – original draft, Writing – review & editing, Conceptualization, Software. CZ: Writing – review & editing, Software, Validation. LY: Writing – review & editing. LH: Writing – review & editing. ZM: Writing – review & editing, Funding acquisition, Resources, Project administration, Supervision. XF: Writing – review & editing, Funding acquisition, Resources. HZho: Writing – review & editing. YT: Resources, Writing – review & editing.
